# Approaches to Unmask Functioning of the Uncultured Microbial Majority From Extreme Habitats on the Seafloor

**DOI:** 10.3389/fmicb.2022.845562

**Published:** 2022-03-29

**Authors:** Stefanie Böhnke, Mirjam Perner

**Affiliations:** Geomicrobiology, GEOMAR Helmholtz Centre for Ocean Research Kiel, Kiel, Germany

**Keywords:** hydrothermal vents, uncultured microbial majority, microbial dark matter, functional metagenomics, *in situ* technologies, activity-based screening, novel enzymes

## Abstract

Researchers have recognized the potential of enzymes and metabolic pathways hidden among the unseen majority of Earth’s microorganisms for decades now. Most of the microbes expected to colonize the seafloor and its subsurface are currently uncultured. Thus, their ability and contribution to element cycling remain enigmatic. Given that the seafloor covers ∼70% of our planet, this amounts to an uncalled potential of unrecognized metabolic properties and interconnections catalyzed by this microbial dark matter. Consequently, a tremendous black box awaits discovery of novel enzymes, catalytic abilities, and metabolic properties in one of the largest habitats on Earth. This mini review summarizes the current knowledge of cultivation-dependent and -independent techniques applied to seafloor habitats to unravel the role of the microbial dark matter. It highlights the great potential that combining microbiological and biogeochemical data from *in situ* experiments with molecular tools has for providing a holistic understanding of bio-geo-coupling in seafloor habitats and uses hydrothermal vent systems as a case example.

## Introduction

The ocean’s seafloor covers ∼70% of our planet’s surface and is vastly underexplored. Through its pivotal role for processing deposited material in marine sediments, the seafloor is critically involved in the extent to which carbon sequestration, nutrient recycling, carbonate dissolution and methane production occur (cf. Middelburg, 2018; LaRowe et al., 2020). Most of the seafloor is in the deep-sea and is hallmarked by hostile conditions, i.e., no light, high pressure, food scarcity, and is mostly characterized by comparatively low turnover rates (Middelburg et al., 1993). Although hydrothermal deep-sea vent ecosystems can be associated with even more extreme conditions, such as high temperatures or the presence of toxic compounds (Perner et al., 2014; McDermott et al., 2018), the emitted inorganic energy sources and chemosynthetic microbes capable of coping with local extreme conditions transform deep-sea hydrothermal vents into hot spots of activity. Venting is also a significant metal source to the ocean, with metal-organic complexation facilitating long-distance transport and potentially impacting primary production in the ocean’s surface (Sander and Koschinsky, 2011; Resing et al., 2015; Fitzsimmons et al., 2017; Ardyna et al., 2019). Additionally, hydrothermal environments are relevant for providing bioactive trace metals (Li et al., 2014; Cohen et al., 2021) and organic carbon (Toner et al., 2009; Bennett et al., 2015; Longnecker et al., 2018), and give insights into the origin of life and its limits (Martin et al., 2008).

Microbes make up most of the total biomass on Earth. However, the majority of prokaryotic cells resist cultivation and remain uncharacterized (Lloyd et al., 2018; Zamkovaya et al., 2021). It is estimated that this uncultured prokaryotic majority, often referred to as microbial dark matter, accounts for up to 91 and 96% of uncultured bacteria and 87 and 96% of uncultured archaea in marine sediments and hydrothermal vents, respectively (Lloyd et al., 2018). Sequencing of prokaryotic (meta)genomes has demonstrated that up to 40% of annotated genes cannot be allocated to a known or predicted function (Baric et al., 2016) and only as little as 16% of ocean metagenomic DNA encoding hypothetical proteins could be linked to proteins with an experimentally verified function (Sunagawa et al., 2015). One way to address this sequence-based limitation is the development of novel computational approaches like, e.g., the CSBFinder-S software. It allows identification of operon structures by inferring conserved synthetic blocks (CSBs), providing a functional context for unassignable enzymes (Svetlitsky et al., 2020).

**FIGURE 1:**
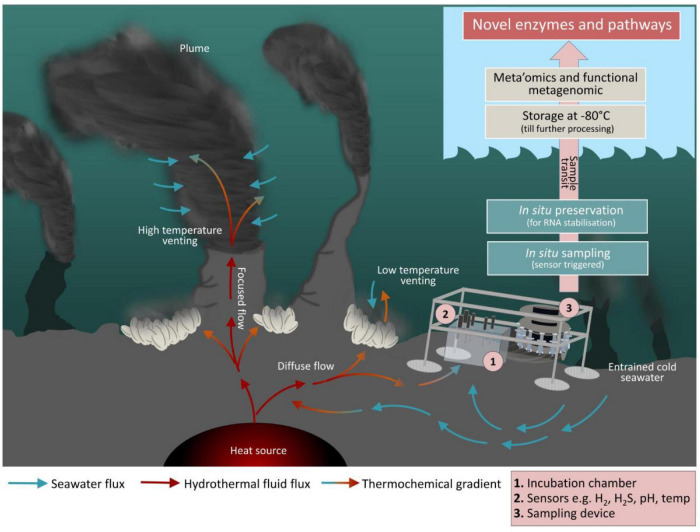
Future perspective for hydrothermal vent *in situ* incubations. A mini chamber lander and the related work flow is shown next to a hydrothermal vent. The mini chamber is equipped with various sensors to measure the local environmental parameters like O_2_, H_2_, H_2_S, pH, temperature, redox potential, and conductivity. Sampling may take place as a time series and/or controlled through the change in certain environmental parameters. Moreover, it is possible to simulate particular what-if scenarios as the syringe samplers may also function as injectors. This allows for manipulation of certain environmental conditions in the chamber as incubation proceeds. The subsamples collected during incubation are filtered and preserved *in situ* using appropriated fixation reagents. Finally, once on board, filters are stored at –80°C until further processing in the home laboratory. Mini chamber lander systems comparable to the here illustrated one have successfully been used to investigate benthic fluxes at the sediment-water interface zone of shallow waters or in the deep-sea (Thoms et al., 2018; Vonnahme et al., 2020; Kononets et al., 2021 and references therein). However, we are not aware of any published work that has reported data generated from the here presented approach where *in situ* incubation at hydrothermal vent environments with sensor-triggered sampling, *in situ* preservation, and subsequent microbiological analyses has been combined to elucidate interrelationships and interdependencies between abiotic factors and the biological world.

So far, meta’omics has given us valuable insights into the taxonomic diversity, metabolic potential and gene expression patterns of microbial communities from extreme seafloor habitats (Fortunato and Huber, 2016 and references therein). Albeit, activity-based screening of metagenomic libraries is the only methodology that currently allows detection of entirely novel enzymes from known and unknown microbes for which homologies to known motifs lack and is a promising approach to overcome shortcomings associated with sequence-based strategies (Handelsman, 2004; Böhnke and Perner, 2014; Adam and Perner, 2018; Pushkarev et al., 2018). However, recombinant expression of metagenomic fragments in a surrogate host can be troublesome due to manifold reasons (divergent codon usage, translation, correct folding etc.), often leading to low hit rates that require high screening throughput which is the reason why functional metagenomic approaches might be very time-consuming and cost intensive (Perner et al., 2011b). Another way to study yet uncultured microbes is to perform the corresponding investigations directly in the natural habitat, i.e., *in situ*. Here, the main challenge is not only to further develop sensor technology and to optimize the collection and preservation of sample material, but also to provide technologies that synchronize *in situ* microbiological and geochemical investigations in space and time (Figure 1).

## The Not Yet Cultivated Microbial Majority and Its Potential for Element Cycling at Hydrothermal Vent Habitats

Deep-sea hydrothermal vent environments form along spreading ridges, where hot, highly reduced hydrothermal fluids mix with cold, oxygenated seawater, thereby creating steep thermal and chemical gradients. Chemosynthetic microorganisms exploit this thermodynamic disequilibrium by generating energy through redox reactions potentially fueling autotrophic carbon fixation. Since the discovery of hydrothermal vents (Ballard, 1977; Corliss et al., 1979), great cultivation efforts have been made to describe metabolic activities and physiological properties of respective microbes (reviewed in Dick, 2019). Cultivation is irreplaceable and includes (i) traditional and steadily improved techniques on liquid or solid media (cf. Reysenbach and Götz, 2001; Hansen and Perner, 2015; Zhang et al., 2018; Zeng et al., 2021 and references therein), (ii) gradient tube incubations (Emerson and Moyer, 1997), enrichments (iii) in bio-electrochemical systems (Pillot et al., 2018), (iv) on *in situ* enrichment carriers (Stokke et al., 2020), or (v) possibly–in the near future–even on synthetically grown hydrothermal vents (Barge and White, 2017; Martinez et al., 2019; Sanchez, 2021), and high-pressure laboratory techniques (Kato, 2011). Information from meta’omic data holds great promise to further improve the cultivation success by guiding the development of new cultivation technologies and strategies that are more responsive to the requirements of uncultured lineages (Gutleben et al., 2018). Once strains are in culture, the next step is the generation of a pure culture, but isolation of microbes is far from trivial. Strains often tend to grow in close co-culture with other strains and a variety of different isolation strategies include plating techniques (cf. Sass and Perner, 2020), role-tube isolations (cf. Zeng et al., 2013), dilution to extension approaches (cf. Adam et al., 2021), single cell separation micro tweezer technologies (Fröhlich and König, 2000; cf. Sass et al., 2020), flow cytometry (Ferrari et al., 2012), diffuse chamber incubation (Kaeberlein et al., 2002) etc. These approaches have resulted in the description of some hundred microbial species with hydrothermal origin (Jebbar et al., 2015). Nevertheless, sequence-based metagenome studies disclose a large discrepancy between microbes present in a certain environment and those that are cultivable (Rinke et al., 2013; Hug et al., 2016; Zamkovaya et al., 2021). With respect to hydrothermal systems and marine sediments, this corresponds to 4 and 9% cultured bacteria and 4 and 13% cultured archaea, respectively (Lloyd et al., 2018). Despite technical progress and relentless efforts, hydrothermal vents are still among the ecosystems with particularly high numbers of uncultivated representatives (Lloyd et al., 2018). Meta’omic studies of hydrothermal vent habitats suggest that the functional differences between closely related microbial species or strains are substantial (Hug et al., 2016; Dombrowski et al., 2017). This highlights an unprecedented potential for various new metabolic pathways and enzyme functions hidden among the non-cultured majority of hydrothermal vent microbes (Zamkovaya et al., 2021). In order to cope with the ever-increasing amount of sequence information and to prevent the gap between physiological and sequence-based information from widening, (high-throughput) approaches linking sequences with functions urgently need to be further developed and advanced.

## Metagenomics: Towards Understanding the Metabolic Microbial Network

Metagenomics refers to the entire genetic information of a given ecosystem (Handelsman et al., 1998). The original metagenomic approach was based on sequence- or function-based screening of metagenomic libraries that contained cloned environmental DNA (Lam et al., 2015). In 2004, the large marine whole genome shotgun sequencing project of the Sargasso Sea, provided, for the first time, a glimpse into the complex microbial community compositions of ocean habitats (Venter et al., 2004), pioneering future metagenome projects. The progress in next generation sequencing technologies has been rapid and together with bioinformatic tool development has allowed the subfields of metatranscriptomics and metaproteomics to further revolutionize meta’omic research (Simon and Daniel, 2011), as gene expression and protein profiles now enable insights into active metabolic processes and functional adaptations (Wilmes et al., 2015; Shakya et al., 2019). In this context, the term functional metagenomics has popped up frequently. This is rather misleading, as this term was originally used for function-based screening of metagenomic libraries seeking specific enzyme activities or valuable compounds (Handelsman, 2004). In the following we use the term functional metagenomics as it was initially coined.

However, high-throughput meta’omic approaches nowadays result in the rapid accumulation of DNA, RNA, and protein sequences, but current databases only allow the assignment of candidate functions based on homologs of already known motifs (Daniel, 2005). Thus, the vast majority of predicted enzyme functions have never been experimentally proven. Indeed, about one-third of the genes found in genomes of cultured and uncultured prokaryotes cannot even be assigned a predicted function due to the lack of homologies (Lloyd et al., 2018). One possible approach suited to verify if a predicted function is true is to clone and express targeted genes in a surrogate host (Yang et al., 2016; Danso et al., 2018; Oppermann et al., 2019). However, one major drawback of this strategy is that the original gene proximity and thus relevant chaperones, transcriptional regulators and/or activators are missing, likely causing corresponding gene products to remain inactive (Böhnke and Perner, 2017). Although the use of large insert metagenomic libraries has the potential to counteract some of these challenges, problems with heterologous gene expression in the surrogate host, e.g., failed gene expression and incorrect post-transcriptional processing, remain one of the major limitations of functional metagenomic approaches (Perner et al., 2011b; Johnson et al., 2017). The use of custom expression strains, alternative vector systems, ionic liquids, or even *in vitro* recombinant transcription systems are promising techniques to mitigate these shortcomings (Lam et al., 2015; Kinfu et al., 2017; Mital et al., 2021).

Implementing a functional metagenomic approach requires two further major challenges to be overcome. First, there is the need to construct metagenomic libraries whereby isolation of high-quality environmental DNA is critical for successful cloning. The second major bottleneck is the often very time consuming and tedious establishments of high-throughput screening methods. A large range of biotechnologically motivated screening technologies for identifying novel biocatalysts or valuable biomolecules with industrial, commercial, clinical or bioremediational applications from uncultured microbes has identified proteases, oxidoreductases, esterases, amylases, phosphatases, chitinases, cellulases, glycosyltransferases, and decarboxylases (cf. Perner et al., 2011b; Rabausch et al., 2013; Berini et al., 2017; Johnson et al., 2017). However, functional metagenomic approaches with ecologically oriented objectives are extremely rare; although some enzymes discovered out of a biotechnological interest may also offer insights into ecologically relevant metabolic processes. Recently, one of the few purely ecologically and biogeochemically motivated functional screening approaches available targeted the distribution of active ribulose-1,5-bisphosphate carboxylases (RubisCO) at different hydrothermal vents (Böhnke and Perner, 2019). The study managed to place the identified RubisCOs (and respective uncultivated microbes) into an ecological context and demonstrated some possible RubisCO-protein interactions with neighboring gene products (Böhnke and Perner, 2017). As part of this work, some of the previously annotated “hypothetical proteins with unknown functions,” could be assigned the probable role as RubisCO transcriptional regulators and post-translational activators or repressors.

Additionally, a second ecologically motivated function-based screen was developed that also targets RubisCO activity (Varaljay et al., 2016). Since Varaljay et al. (2016) used a different host-vector system, this heterologous complementation based functional metagenomic screen likely expands the spectrum of detectable active RubisCOs (Varaljay et al., 2016). Another ecologically and biogeochemically motivated functional metagenomic approach focused on hydrogenase activities (Adam and Perner, 2017). The screening detected three H_2_-uptake expressing active metagenomic clones without any known hydrogenase-encoding genes or motifs on their DNA insert (Adam and Perner, 2018) suggesting novel hydrogenases. The discovery of heliorhodopsin, a globally abundant and widely distributed light-sensing rhodopsin, has also been enabled by functional metagenomics (Pushkarev et al., 2018). These studies highlight the tremendous diversity of currently unknown dark matter proteins and underline the urgent need for developing more novel screening methods for targeting specific enzymatic activities of unknown organisms. This methodology allows a window into the metabolic network of the uncultured microbes and their catalytic ability in biogeochemical cycling of key elements.

## Current Challenges and Future Perspectives for *in situ* Technologies at the Seafloor

Marine microbial communities hold a central role as drivers of major biogeochemical processes, impacting ecosystem functioning far beyond the oceans (Falkowski et al., 2008). Research into these microbial consortia and the processes they mediate is, however, often constrained by technical capabilities, as is particularly evident in deep-sea research (Fortunato et al., 2021). Thus, sampling hard accessible deep-sea environments is already a technical and logistical challenge, requiring the development of specialized underwater devices (Liang et al., 2021; Paulus, 2021). Over the past decades a variety of ocean deployable sampling instrumentation have been developed (McQuillan and Robidart, 2017). Yet, only a few of them are suited to retrieve samples from extreme deep-sea habitats and are able to withstand the high pressures and corrosive hot fluids (Reysenbach and Götz, 2001; Liang et al., 2021). Transporting the samples from the deep-sea through the water column to the research vessel laboratory poses further inherent limitations as the samples are exposed to physico-chemical changes (e.g., changes in pressure, temperature, light, pH, redox state etc.) altering the compositions and thus biasing subsequent analysis (Edgcomb et al., 2016). Chemical composition of sampled hydrothermal fluids can change dramatically if *in situ* pressure is not maintained, resulting in degassing and the loss of volatile species and distorting microbial activities and metabolic rates (McNichol et al., 2016). Gas-tight sampling devices are used to mitigate this effect and prevent outgassing (Seewald et al., 2002; Butterfield et al., 2004; Miyazaki et al., 2017; Wu et al., 2018; Garel et al., 2019; Wang et al., 2020). A long and often variable lag time during ascent may change redox reactions, introducing artifacts in subsequent analyses despite the usage of pressure maintaining sampling devices (Fortunato et al., 2021). Once on board, the samples are subjected to atmospheric pressure for *ex situ* filtration, likely causing cell lyses and release of RNA and DNA molecules (Edgcomb et al., 2016). Extracellular DNA and RNA from lysed cells can only partially be bound and recovered by filtration (Liang and Keeley, 2013), thereby losing parts of the unknown microbial community. Unpreserved biological material is also very labile and starts to degrade within minutes (RNA and proteins) or hours to days (cells and DNA), further biasing samples (Ottesen, 2016). Indeed, a comparative study of *in situ* and shipboard RNA stable isotope probing (RNA-SIP) experiments showed that microbial communities are significantly affected by the effects of depressurization and sample processing delays, resulting in a shift of the community structure and metabolic function (Fortunato et al., 2021). *In situ* preservation is one approach that has successfully been used to overcome limitations associated with sample transit (Edgcomb et al., 2016; Fortunato et al., 2021). But devices designed for filtration and integrated subsequent preservation are still rare (reviewed in Ottesen, 2016). They include the Suspended Particulate Rosette V2 (SUPR-V2) System (Breier et al., 2014), the Biological Osmo Sampling System (BOSS) (Robidart et al., 2013), and the Fixation Filter Unit (FF3) (Taylor et al., 2015).

The more information on habitat specific physicochemical characteristics available, the more value can be deduced from generated meta’omic datasets. This is essential if aiming to understand the role of microbes for ocean ecosystem functioning. Deep-sea sensors are efficient tools for observing local geochemistry, allowing real-time monitoring of certain key chemical variables such as pH, dissolved H_2_, H_2_S, CH_4_, CO_2_, and dissolved inorganic nutrients (Luther et al., 2001; Moore et al., 2009; Petersen et al., 2011; Wankel et al., 2011; Perner et al., 2013; Daniel et al., 2020; Gros et al., 2021; Liang et al., 2021; Mowlem et al., 2021). However, technical limitations require that various chemical parameters still have to be determined *ex situ* (Mowlem et al., 2021). Although *in situ* filtration allows reduction of chemical alteration caused by precipitation and/or adsorption of some dissolved elements during transit from the seafloor to the ship’s research laboratory, it is evident that the most representative data on deep-sea fluid chemistry would be provided by direct *in situ* measurements (Sievert and Vetriani, 2012; Cotte et al., 2015). Thus, future efforts must be directed toward further advancing existing sensors (more precision, robustness, serialization and standardization) and establishing novel sensor technologies.

**FIGURE 2:**
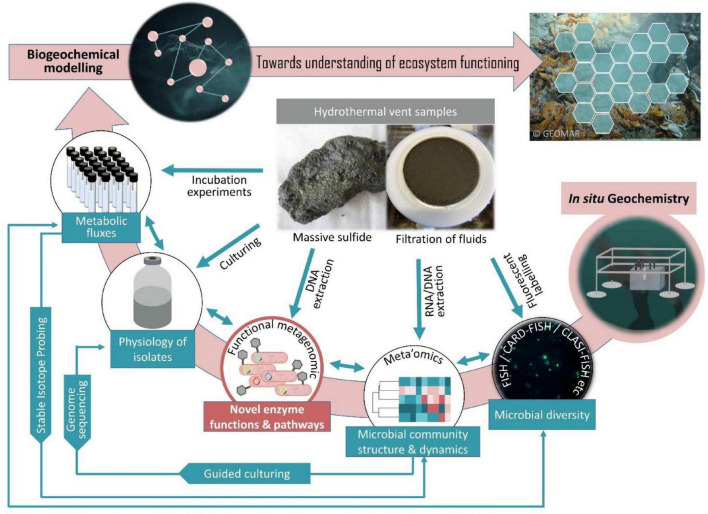
A holistic approach to study the structure and function of microbial consortia in hydrothermal vents. The overview shows the approaches that should be considered and combined, if aiming at an omni-directional insight into hydrothermal vent ecosystem functioning, without neglecting the yet uncultivable majority of microorganisms.

Technological advances in the past decade have enabled the development of a limited set of samplers capable of performing *in situ* experiments directly in the deep-sea, pioneering future biogeochemical studies in deep-sea habitats. Respective devices have successfully been used to perform *in situ* tracer incubations (Edgcomb et al., 2016), RNA-SIP experiments (Fortunato et al., 2021), molecular analytical techniques (Scholin et al., 2017), and extraction of organic compounds (Grandy et al., 2020). This has impressively demonstrated that *in situ* experiments can provide a window into the seafloor microbial consortia, metabolic mechanisms and transformations. To obtain a more complete understanding of microbial community dynamics, functions and influences on ocean processes, microbiology and geochemistry must be sampled simultaneously. Automated mini chamber lander systems have a great potential as they allow time series sampling in response to changes of environmental conditions, e.g., O_2_, H_2_S etc. (Figure 1). Furthermore, the possibility of injecting selected chemical compounds into the *in situ* incubation chamber could be used to simulate different what-if-scenarios. Thereby they can contribute to forecasting potential climate change impacts on the deep-sea microbes and the biogeochemical processes they mediate. Embedded in a holistic approach (Figure 2), *in situ* microbiological and biogeochemical analyses conducted in spatial and temporal proximity to each other can provide a more comprehensive picture of what features influence overall biogeochemical fluxes. This in turn improves the basis for building predictive models of how deep-sea microbial consortia contribute to global biogeochemical cycles.

Biogeochemical modeling is successfully used to determine (i) element flux rates of trace metals like, e.g., Fe, Mn, Ni, Cu, Co, Cd and Zn (reviewed in Homoky et al., 2016; cf. Somes et al., 2021), (ii) particulate organic material (POM) reactivity (reviewed in Lessin et al., 2018), (iii) thermodynamics (cf. Perner et al., 2011a) and (iv) energetics (cf. Böhnke et al., 2019), there by enhancing our theoretical and quantitative understanding of microbial and geochemical interactions (Dick, 2019). Only a few biogeochemical models have been established in recent years that allow the linkage between microbial biogeochemical rate measurements and meta’omic data, making key unknown physiological parameters, such as kinetic properties, transcription and translation rates, and mRNA and protein degradation rates recognizable (Reed et al., 2014; Louca et al., 2016). Such models have great potential and hold promise to unprecedented predictions about the role of ubiquitous microorganisms in mediating global element cycling.

## Conclusion

The current understanding of the contribution of seafloor microbes to global biogeochemical cycles, metabolic fluxes and ecosystem functions is primarily aligned with what we known from culturable microbes. The cultured microbes, however, only represent a minor fraction of the total microbial vent community. This shows that our current understanding is vastly incomplete. Cultivation-independent approaches including *in situ* technologies, biogeochemical rate measurements, functional metagenomics, meta’omics, and biogeochemical modeling are promising tools that have already been used to effectively complement cultivation-dependent methods. Clearly, no single technology will provide full access to the vast potential of novel metabolic pathways hidden among the majority of uncultured microorganisms. The great challenge, but also the most promising approach for the future, can only lie in harnessing the strengths of available cultivation-dependent and cultivation-independent tools and smartly combining them in a holistic multidisciplinary approach. Here, continuing the development of existing *in situ* technologies and experimentation, but also the establishment of completely new ones, is of major importance and will significantly drive progress toward opening the window into previously inaccessible microbial physiologies of the microbial dark matter.

## Author Contributions

SB and MP wrote the manuscript. Both authors contributed to the article and approved the submitted version.

## Conflict of Interest

The authors declare that the research was conducted in the absence of any commercial or financial relationships that could be construed as a potential conflict of interest.

## Publisher’s Note

All claims expressed in this article are solely those of the authors and do not necessarily represent those of their affiliated organizations, or those of the publisher, the editors and the reviewers. Any product that may be evaluated in this article, or claim that may be made by its manufacturer, is not guaranteed or endorsed by the publisher.
